# Sensitivity Enhancement of Curvature Fiber Sensor Based on Polymer-Coated Capillary Hollow-Core Fiber

**DOI:** 10.3390/s20133763

**Published:** 2020-07-05

**Authors:** Luis A. Herrera-Piad, Iván Hernández-Romano, Daniel A. May-Arrioja, Vladimir P. Minkovich, Miguel Torres-Cisneros

**Affiliations:** 1 Electronics Department, DICIS, Universidad de Guanajuato, Carretera Salamanca-Valle de Santiago km 3.5 + 1.8, Salamanca 36885, Mexico; la.herrerapiad@ugto.mx (L.A.H.-P.); torres.cisneros@ugto.mx (M.T.-C.); 2 CONACYT-Electronics Department, DICIS, Universidad de Guanajuato, Carretera Salamanca-Valle de Santiago km 3.5 + 1.8, Salamanca 36885, Mexico; 3 Fiber and Integrated Optics Laboratory (FIOLab), Centro de Investigaciones en Óptica A.C., Aguascalientes 20200, Mexico; darrioja@cio.mx; 4 Centro de Investigaciones en Óptica A.C., Calle Lomas del Bosque 115, León 37150, Mexico; vladimir@cio.mx

**Keywords:** curvature sensing, ARROW guidance, PDMS covering, capillary hollow-core fiber

## Abstract

In this paper, we propose and experimentally demonstrate a simple technique to enhance the curvature sensitivity of a bending fiber optic sensor based on anti-resonant reflecting optical waveguide (ARROW) guidance. The sensing structure is assembled by splicing a segment of capillary hollow-core fiber (CHCF) between two single-mode fibers (SMF), and the device is set on a steel sheet for measuring different curvatures. Without any surface treatment, the ARROW sensor exhibits a curvature sensitivity of 1.6 dB/m^−1^ in a curvature range from 0 to 2.14 m^−1^. By carefully coating half of the CHCF length with polydimethylsiloxane (PDMS), the curvature sensitivity of the ARROW sensor is enhanced to −5.62 dB/m^−1^, as well as an increment in the curvature range (from 0 to 2.68 m^−1^). Moreover, the covered device exhibits a low-temperature sensitivity (0.038 dB/°C), meaning that temperature fluctuations do not compromise the bending fiber optic sensor operation. The ARROW sensor fabricated with this technique has high sensitivity and a wide range for curvature measurements, with the advantage that the technique is cost-effective and easy to implement. All these features make this technique appealing for real sensing applications, such as structural health monitoring.

## 1. Introduction

Fiber optics sensors (FOS) are attractive to the scientific community and industry due to their intrinsic properties, such as small size, lightweight, corrosion resistance, immunity to electromagnetic interference, and high sensitivity. Several FOS have been assembled using specialty fibers such as photonic crystal fiber (PCF) [1,2], capillary hollow-core fiber (CHCF) [3], microfiber [4], D-shaped fiber [5], fiber Bragg grating (FBG) [6,7], long-period fiber grating (LPBG) [8], tilted fiber Bragg grating (TFBG) [9], and multicore optical fiber [10]. Recently, the fabrication of FOS using CHCF has been increased due to its easy fabrication and low-cost. Using this specialty fiber it has been possible to implement Mach-Zehnder [11], Fabry-Pérot [12,13], and Sagnac [14,15] interferometers for sensing applications, as well as sensors based on the multimode interference effect [16] and the anti-resonant reflecting optical waveguide (ARROW) guidance [17]. In the last few years, the use of CHCF to observe ARROW guidance has become an appealing technique to construct FOS, in this case, light is guided with relatively low loss by the anti-resonant FP resonator formed by the silica ring in the air. Therefore, the fabrication of ARROW devices is quite simple and requires only the splicing of a section of CHCF between two single-mode fibers (SMF). Since the ARROW guidance mechanism exhibits a transmission spectrum with periodic dips, we can use it as a sensor by following either wavelength shifts or intensity changes (based on the FOS design). Different parameters have been measured using ARROW sensors such as temperature [18], pressure [19,20], magnetic field [21,22,23], relative humidity [24], torsion [25], liquid level [26], displacement [27], and curvature [28] just to mention a few. In particular, measuring curvature is a crucial task that needs to be implemented in different areas such as robotic arms [29] and structural health monitoring [30], which includes the monitoring of bridges, buildings, airplanes, and other structures. In the above applications, fiber optic curvature sensors have been widely employed due to their easy integration, portability, and multiplexing capabilities. There has been a particular interest in curvature sensors based on monitoring intensity changes on observed dips in the transmitted spectral response, and ARROW devices based on CHCF exhibit that feature. For that reason, W. Ni et al. [31] demonstrated a fiber sensor capable of measuring temperature and curvature using a segment of single hole twin eccentric core fiber, exhibiting a curvature sensitivity of −1.54 dB/m^−1^ in a range from 0.94 to 2.1 m^−1^. R. Gao et al. [32] fabricated a directional curvature sensor by infiltrating two symmetric air holes in a hollow-core PCF. The curvature sensor was able to measure two appositive directions with sensitivities of 4.86 and 4.84 dB/m^−1^ in a curvature range from 0 to 0.88 m^−1^. S. Wang et al. [28] implemented a curvature sensor with high sensitivity (−15.33 dB/m^−1^) in the range from 3.63 to 4.69 m^−1^. A dual parameter FOS for curvature and temperature was proposed by H. Cheng et al. [33], demonstrating a curvature sensitivity of −4.28 dB/m^−1^ within a curvature range from 10.72 to 11.60 m^−1^. F. Zhao et al. [34] fabricated a curvature sensor for monitoring power grid wires, and its curvature sensitivity was 3.414 dB/m^−1^ in a range from 0 to 2.122 m^−1^. It is important to highlight that the curvature sensitivity and the curvature range are two essential parameters to consider when designing FOS for curvature applications. In many cases, as we noticed in the above FOS configurations, a tradeoff between them occurs, and depending on the application one of them is selected as an important element for sensing.

Here, we present a simple technique to enhance the sensitivity of a curvature sensor based on the ARROW structure. Our approach relies on the fact that by reducing the refractive index (RI) contrast between the core and cladding of a waveguide, the induced optical losses are increased when the waveguide is bent. The above is also true in the case of ARROW waveguides, such as the ones fabricated with CHCF, since the ring-cladding will experience some losses when it is bent. Therefore, when we cover the CHCF with polydimethylsiloxane (PDMS), this will significantly reduce the RI contrast between the silica and air, which should enhance the induced losses as a function of the applied curvature. It is important to highlight that after splicing the CHCF to the SMF, we do not need to perform any particular procedure rather than carefully covering a specific length of the CHCF. Our experimental results confirm that our ARROW sensor covered with PDMS exhibits an enhanced curvature sensitivity of −5.62 dB/m^−1^ within a sensing range from 0 to 2.68 m^−1^. The observed sensitivity corresponds to an enhancement of 3.5 times as compared to the sensitivity of the ARROW curvature sensor without PDMS. We should also mention that the temperature sensitivity of the ARROW sensor with PDMS is low (0.038 dB/°C), which demonstrates that the curvature measurements are not affected by temperature fluctuation.

## 2. Principle Operation, Fabrication Method, and Experimental Setup

### 2.1. Operation Principle

The ARROW mechanism that occurs in a CHCF is a well-known effect that has been used for sensing many physical parameters. ARROW devices are constructed, as we mentioned before, by splicing a segment of CHCF between two SMF. In this configuration, the lead-in fiber launches light in the central hollow region of the CHCF (air) and, as the RI of hollow-core is lower than that of the ring-cladding (silica) region, the core modes radiate light through the ring-cladding. The ring-cladding region can be thought of as an FP resonator where the wavelengths that are close to the resonant condition will go through the ring-cladding region and leak out from the CHCF. Such wavelengths are related to the periodic lossy dips observed in the transmitted spectra. On the other hand, the wavelengths that do not satisfy the resonant condition will be internally reflected, confined in the hollow-core, and these wavelengths will be guided through the hollow-core until they reach the lead-out fiber. These confined wavelengths are related to the periodic peaks observed in the transmission spectrum. The reflected intensity from the ring-cladding (FP) that is transmitted in the hollow core of the CHCF can be given as [26]

(1) $$I_{r}={\left|\frac{r_{1}+r_{2}e^{i\delta }}{1+r_{1}r_{2}e^{i\delta }}E_{0}\right|}^{2},$$

where $r_{1}$ and $r_{2}$ are the reflection coefficients of the interfaces between air-core/ring-cladding and ring-cladding/surrounding-medium. $E_{0}$ is the amplitude of the incident light. The reflected adjacent rays from the FP that come back to the air-core of the CHCF have a constant optical path difference, which is given by $\delta =\left(4\pi /\lambda \right)ndcos\left(\theta_{2}\right)+\;\pi$, where λ, n, d, and $\theta_{2}$ are the wavelength in free space, the RI of the ring-cladding, the thickness of the ring-cladding, and the refraction angle at the interface between air-core/ring-cladding. From Equation (1) it is easy to realize that the periodic dips depend on the RI and the thickness of the ring-cladding. On the other hand, the contrast of the lossy dips is related to the reflection coefficients $r_{1}$ and $r_{2}$; if these two coefficients are equal, the contrast of lossy dips will be high. This contrast value can be increased by using a longer segment of the CHCF because this increases the number of reflections of propagation light and produce stronger interference. One disadvantage of using a longer segment of CHCF is that the optical power suffers from higher losses. In the end, it is a tradeoff between the contrast of the lossy dips and signal attenuation, as many authors have been shown [24,34,35]. According to Equation (1), if $r_{2}$ decreases the contrast of the lossy dips will be reduced, which can be achieved by starting to cover the outer part of CHCF by a liquid or a polymer. This interesting feature has been used to develop liquid level sensors [26,36], in which the lossy dips disappear when the liquid completely covers the length of the CHCF. The vanishing of this interferometric signal shows that the presence of the material surrounding the CHCF inhibits the leakage of the resonant wavelengths. Therefore, proper selection of the CHCF dimensions provides a periodic spectrum which is adequate for sensing applications.

FOS are used as curvature sensors because when the fiber is bent, they experience induced stress that is directly proportional to the radius of curvature. Under a bending condition, the outer and inner region of the optical fiber (OF) will experience tensile and compressive stress, respectively. The induced stress will modify the OF refractive index (RI) distribution via stress-optic effects [37], and this can disturb the guiding properties of the fiber. For instance, bending will modify the shape of the guided mode that effectively moves toward the outer region of the OF, which will increase the losses of the guided mode [38,39,40]. Although such an effect is detrimental for telecom applications, some curvature sensors take advantage of this effect, and different curvature FOS have been demonstrated as we mentioned before [28,41,42,43]. In the case of ARROW devices based on CHCF, bending losses also occur, and light that travels in the hollow-core and the ring-cladding leaks out as radiative light, increasing the propagating losses. Therefore, bending losses cause intensity changes in the transmitted spectrum, allowing us to monitor curvature following intensity changes in any of the periodic lossy dips. 

It is essential to highlight that, in the case of bending losses, the RI contrast between the OF core and cladding plays an important role. For instance, if the RI contrast between the OF core and cladding is low, the induced bending losses will be higher, and vice versa. Therefore, a simple way to increase the sensitivity of an ARROW curvature FOS is by reducing the RI contrast between the ring-cladding region of the CHCF and the air surrounding the CHCF. The RI contrast can be easily reduced by covering or embedding the CHCF in a polymer with a RI value closer to that of the silica. We use the well-known polydimethylsiloxane (PDMS) polymer to cover the CHCF section in order to enhance the sensitivity of an ARROW curvature FOS. Since the PDMS has a RI that is close to that of the silica, we need to optimize the CHCF length that can be covered with PDMS to keep the resonances in the transmitted spectrum. This optimization is crucial to obtain the highest sensitivity of the ARROW curvature sensor while preserving the sensor’s ability to monitor curvature and will be explained in detail in the following sections.

### 2.2. Fabrication Method

**Figure 1:**
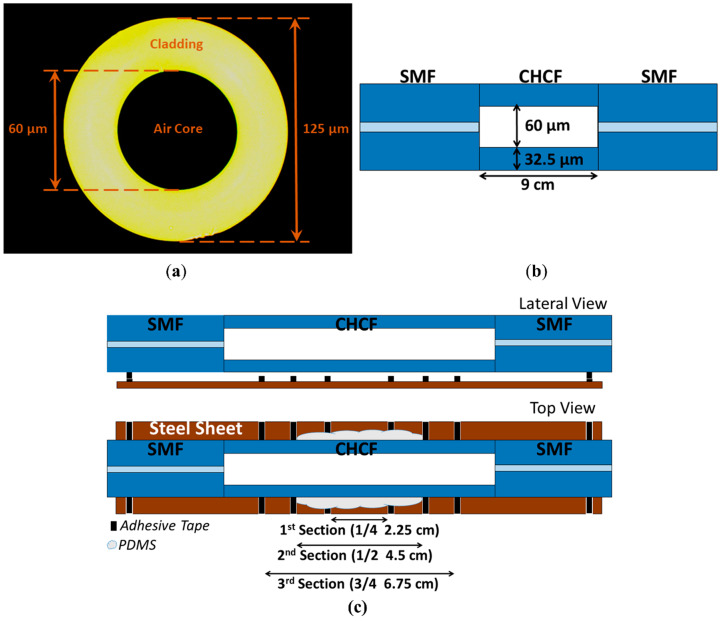
(**a**) Cross-section of the CHCF; (**b**) Sketch of the ARROW sensor structure; (**c**) Lateral view (without polymer), top view (second section covered with polymer).

Figure 1a shows a picture of the cross-section of the CHCF taken by an optical microscope. The inner and outer diameters of the CHCF are 60 and 125 μm, respectively. This CHCF does not have acrylate protection and was fabricated in the Fiber Optic Fabrication Laboratory at *Centro de Investigación en Óptica, Mexico*. The SMF used in this experiment was a commercial SMF-28e+ (the cladding and core diameters are 125 and 8.2 µm, respectively) [44]. The sensor was constructed by splicing a 9 cm long segment of CHCF between two SMF, see Figure 1b. It should be mentioned that different lengths of CHCF were characterized since the length of the CHCF is related to the contrast of the lossy dips and also signal attenuation, as was mentioned in the last section. By using a 9 cm long segment of CHCF, we found the best contrast of the lossy dips and satisfactory optical signal power to detect power variation accurately. Due to their different geometry, splicing CHCF and SMF with a standard splicing program introduces high losses. We developed a particular program using a Fitel splicer (model s179) to optimize the splicing between CHCF and SMF and reduce the splicing losses. The program first performs an automatic cladding alignment before the splicing. The splicing parameters used in the program were 15 mA of arc power, 10 µm of gap (distance between end face of SMF and CHCF), and 300 ms of arc fusion time. After the fabrication of the sensing structure, the CHCF was cleaned by acetone with the help of some swabs. It is noteworthy that the structure does not need any further surface treatment before pouring the polymer. 

The polymer PDMS was used to enhance the ARROW curvature sensor because it has several properties that are ideal in our application, such as a lower RI than the silica (~1.42 at 1550 nm), it can be easily bended after curing, and in general is not too expensive. In order to investigate the performance of the fabricated ARROW device as a curvature sensor, it was fixed on a steel sheet with the help of adhesive tape, as shown in Figure 1c. Considering that the RI difference between silica and PDMS is small, we need to identify the maximum CHCF length that can be covered with PDMS without observing detrimental effects on the ARROW spectral response, as it was mentioned in the last part. The PDMS (RI of 1.42 at 1550 nm) is prepared by pouring a 10:2 ratio of the silicone elastomer and curing agent and mixing them for a couple of minutes. This mixture is allowed to rest for 45 min, which is enough time for releasing air bubbles in the polymer. After covering the outer part of the CHCF with PDMS, the device was placed on a hot-plate and heated up at 80 °C for 10 min to cure the polymer. It must be pointed out that the thickness of the polymer layer was 2 mm, and the sensor was set in the middle of this polymer layer (this position inside the material is called the neutral axis). This axis does not suffer any elongation when the segment is bent [30]. We decided to put the sensor in this position to guaranty that it does experience any strain due to the polymer. The thickness of the PDMS film is ensured by assembling a 2 mm thick cavity on the region to be covered with PDMS. After pouring the PDMS, any small air bubble is removed during the single-step curing process, which provides a uniform and homogeneous PDMS film. At this point, the sensor is ready to measure its curvature sensitivity. In order to control the different CHCF lengths that are covered with PDMS, we used tape as guides to separate four sections in the CHCF, see lateral and top views in Figure 1c. The tape only indicates the sections, and it does not have contact with the CHCF. It is important to mention that the first section corresponds to one-quarter of the CHCF length; the second section is one half of the CHCF length, the third section is three-quarters of the CHCF length, and the fourth section is the entire CHCF length.

**Figure 2:**
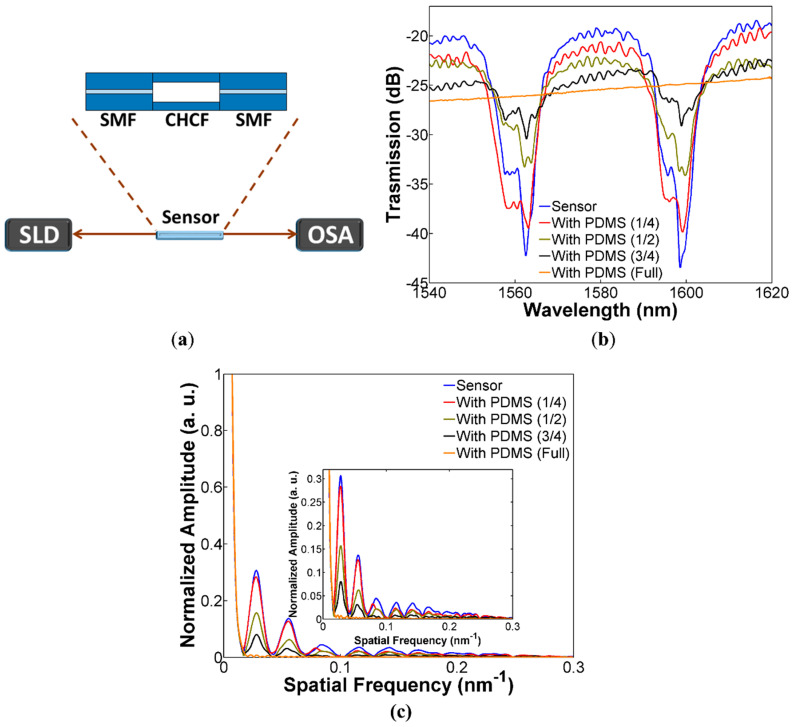
(**a**) Experimental setup for measuring the ARROW sensor spectral response; (**b**) Spectral response of the ARROW sensor, including the bare sensor (without PDMS) and different sections of CHCF covered with PDMS; (**c**) FFT of the transmission spectra.

The output spectral response of the device, for different CHCF lengths covered with PDMS, was analyzed by using the setup shown in Figure 2a. In the setup, we use a superluminescent diode (SLD) as the broadband source (SLD-1550S-A40, Thorlabs, Newton, NJ, USA), which is centered at 1550 nm and with a measurable bandwidth of 100 nm. The optical signal transmitted through the ARROW curvature sensor is acquired by an OSA (MS9740A, Anritsu, Kanagawa, Japan) to study the effects of the PDMS applied to the CHCF. As shown in Figure 2b, the ARROW curvature sensor without PDMS exhibits a spectral response with periodic lossy dips as described by the ARROW guiding mechanism that was previously mentioned. It must be pointed out that a high fringe contrast better than 23 dB was obtained by the sensor without PDMS (blue line) and the fringe contrast generated by the first, second, third, and fourth sections covered with PDMS was 21, 12, 6, and 0 dB, respectively. As the CHCF was covered with longer sections of PDMS, the fringe contrast gradually decreases, as shown in Figure 2b. This experiment corroborates what was said in Section 2.1, covering longer sections of CHCF with PDMS (decreasing $r_{2}$) generates low fringe contrast of the lossy dips. Another interesting feature that is observed in all the transmission spectra is the small ripples that can be produced by two reasons. One is related to the slight variation of the thickness of the ring-cladding due to the manufacturing process or by a small deformation during the fabrication process of the sensor [28,35]. The other is related to the modal interference between the core mode and the cladding modes of the CHCF caused by a mismatch in the fusion splice between SMF and CHCF [28,36]. One way to determine how many modes propagates in the CHCF is by taking the Fast Fourier Transform (FFT) of the transmission spectra, see Figure 2c. It should be mentioned that at least nine modes besides the fundamental mode propagate in the CHCF. When the CHCF was covered entirely with PDMS, only the fundamental mode keeps propagating in the air-core of the CHCF and the other modes (seemingly cladding modes) disappear. The amplitude of cladding modes decreases as the CHCF was covered with longer sections of PDMS. 

A test was carried out to evaluate the performance of this structure as a curvature sensor, since we were monitoring amplitude changes in the lossy dips and the contrast of the signal generated by the third and fourth sections was low, these sections were not characterized for curvature measurements.

### 2.3. Experimental Setup

**Figure 3:**
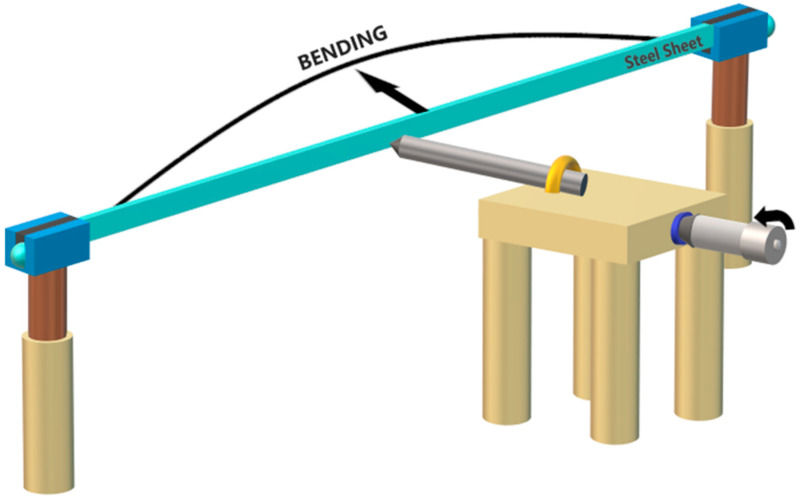
Schematic of the experimental setup for performing curvature measurements.

The experimental arrangement shown in Figure 3 was used to study the performance of the ARROW sensor for measuring different curvatures. The ends of the steel sheet were set on two square grooves, and each square groove was attached to a rotatory system that was fixed to a post. We should highlight that the groove spacing was bigger than the steel sheet thickness, allowing free movement of the steel sheet. The previous approach guarantees that the sensor does not suffer any strain during the curvature experiment. For a more detailed explanation of the setup, see Ref. [41]. A metal screw with a pointed head was mounted in a translation stage and was used to push and curve the steel sheet at curvatures from 0 to 2.68 m^−1^. A SLD was used to launch light to the ARROW sensor through the lead-in SMF, and the output spectrum was acquired using an optical spectrum analyzer (OSA). 

## 3. Results and Discussion

### 3.1. Curvature Measurements without PDMS

**Figure 4:**
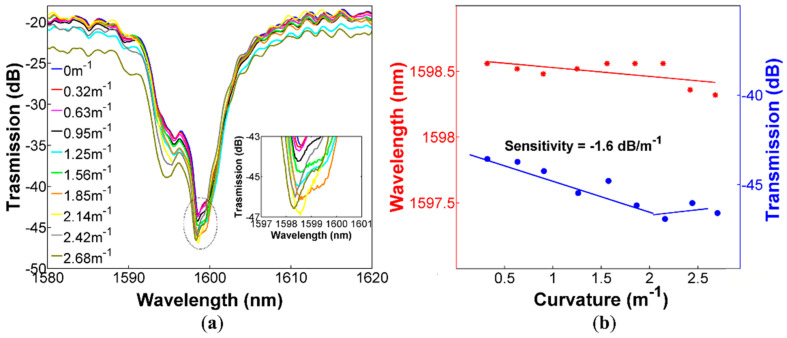
ARROW sensor without PDMS (**a**) Spectral response of the sensor for curvatures from 0 to 2.68 m^−1^; (**b**) Dip wavelength shift and transmitted intensity changes as a function of the applied curvature.

It is important to mention that we are interested in observing the net effects of incorporating the PDMS on the ARROW sensor and eliminate any other potential contribution that could enhance its sensitivity. Thus, the experiment started by first characterizing the device without the polymer, and the results are presented in this section. The transmitted spectral response of the ARROW sensor without PDMS at different curvatures are shown in Figure 4a, and the dip around 1600 nm was chosen to perform the measurements. As can be observed, the curvature changes produce a variation of the dip transmission intensity and a negligible wavelength shift. Therefore, monitoring of the dip transmission changes allows us to correlate such changes to the applied curvature, see Figure 4b. We should also notice that the transmitted intensity changes exhibit a linear response in the range from 0 to 2.14 m^−1^, as well as a curvature sensitivity of −1.6 dB/m^−1^, as shown in Figure 4b. It is worthwhile noting that the curvature sensitivity is lower than the sensitivities of almost all sensors mentioned in the introduction (without considering the range). Nevertheless, we should also emphasize that we neither modified the CHCF at all nor applied any special fabrication procedure to enhance the sensitivity. In fact, this is the main focus of our work, in which the curvature sensitivity can be increased to a competitive value by using the technique of covering a section of the CHCF; see the next section. Also shown in Figure 4b is the dip wavelength shift as a function of the applied curvature, exhibiting a sensitivity of 0.104 nm/m^−1^, which is low enough to interfere with intensity measurements.

### 3.2. Curvature Measurements with PDMS

**Figure 5:**
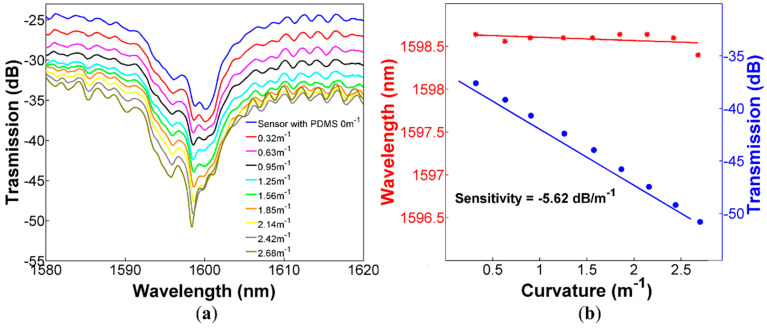
ARROW sensor covered with PDMS (**a**) Spectral response of the sensor for curvatures from 0 to 2.68 m^−1^; (**b**) Dip wavelength shift and transmitted intensity changes as a function of the applied curvature.

The sensor from the last experiment was covered with PDMS, and its response at different curvatures was characterized. Adding the PDMS is the only modification that was made to this sensor, which guarantees that any changes in the performance of this device regarding curvature and temperature measurements are only due to the PDMS. Additionally, since we are not comparing two different devices, we also avoid typical fabrication errors such as different splicing points as well as different lengths of the CHCF during the sensor assembly. We performed curvature measurements in the ARROW devices with the first and second sections covered with PDMS (see Figure 1b). Here we report results only of the ARROW sensor with the second section covered with PDMS (see Figure 1b) since it exhibits the best performance in terms of high fringe contrast, high sensitivity, and wide range. This device was carefully characterized, and its output spectral response at different curvatures is shown in Figure 5a. We can observe that the sensor exhibits significant larger losses when the curvature is increased. In fact, the transmitted intensity changes as a function of the applied curvature improve its linear response over a wider range from 0 to 2.68 m^−1^, while significantly improving the curvature sensitivity to −5.62 dB/m^−1^, see Figure 5b. We should also note that the wavelength shift of the dip near 1600 nm is not significantly modified over the measured curvature range, exhibiting a sensitivity of 0.164 nm/m^−1^.

Based on the above results, we can observe that the curvature sensitivity of the sensor covered with PDMS (half-length of the CHCF) is 3.5 times higher than the sensitivity of the sensor without PDMS, which demonstrates the enhancement in the sensitivity with our proposed technique. 

It is also worth noticing that the CHCF without PDMS exhibits a non-linear behavior, as shown in Figure 4b. Such non-linear response is related to changes in the transmitted spectrum as the CHCF is curved, which results from the multiple beam interference since bending losses are relatively small. On the other hand, when the CHCF is covered with PDMS, the mode confinement is reduced and bending losses are significantly higher. This allows bending losses to be the dominant factor over changes of the transmitted spectrum, and the whole intensity of the transmitted spectrum is reduced, providing a linear response over the entire curvature sensing range, see Figure 5b.

### 3.3. Temperature Sensitivity

**Figure 6:**
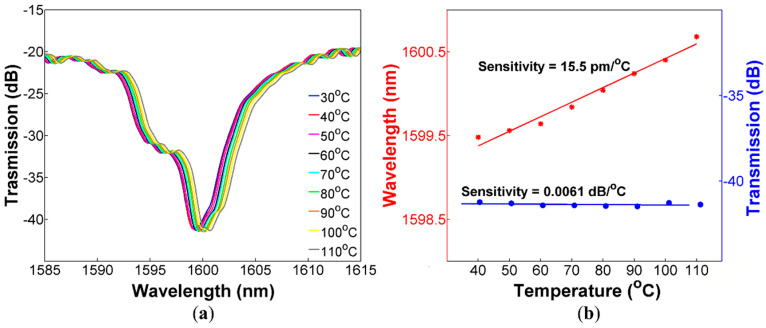
ARROW sensor without PDMS (**a**) Spectral response of the sensor for temperature from 30 to 110 °C; (**b**) Dip wavelength shift and transmitted intensity changes as a function of the applied temperature.

**Figure 7:**
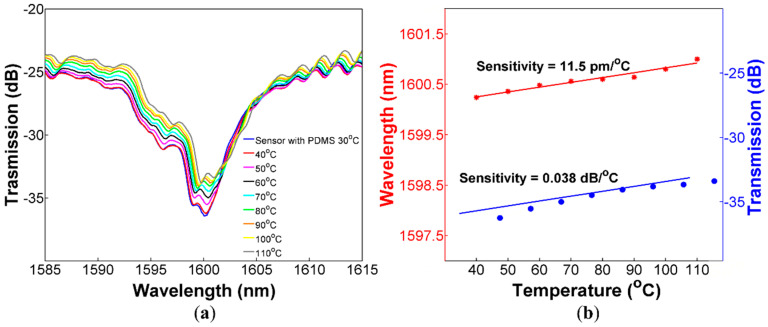
ARROW sensor covered with PDMS (**a**) Spectral response of the device for temperature from 30 to 110 °C; (**b**) Dip wavelength shift and transmitted intensity changes as a function of the applied temperature.

A feature that is desirable for curvature sensors is an athermal response since temperature fluctuations are always present (in real applications), and they can produce measurement errors. The idea of covering a section of CHCF with polymer is to increase the curvature sensitivity of the sensor. However, it is well-known that PDMS polymer has a high thermo-optic coefficient (TOC), and this may cause that our sensors increase its response to temperature. In this section, we describe the experimental procedure to characterize the thermal response of both sensors, with and without PDMS, which allows us to compare their response to temperature variations. The ARROW sensor with and without PDMS was set on a hot plate to investigate their response to temperature changes from 30 to 110 °C in steps of 10 °C. The variations in the spectrum when the temperature was increased for the sensor without PDMS is shown in Figure 6a. We can observe that the dip experiences a wavelength shift to larger wavelengths. Figure 6b shows the temperature sensitivities for the wavelength shift and transmitted intensity changes, which are 15.5 pm/°C and 0.0061 dB/°C, respectively. The same experiment was repeated with the sensor covered with PDMS, exhibiting temperature sensitivities of 11.5 pm/°C (for wavelength shift) and 0.038 dB/°C (for transmitted intensity changes), see Figure 7. Comparing the temperature sensitivity for the transmitted intensity changes of both sensors as a function of temperature, the sensor covered with PDMS is more sensitive than the other, which means that the sensor with PDMS is more affected by temperature fluctuation. Nevertheless, the temperature sensitivity is two orders of magnitude lower than the curvature sensitivity, and it can be neglected during curvature measurements. Furthermore, a cross-sensitivity of 0.0068 m^−1^/°C was calculated, which corroborates negligible interference of temperature changes during curvature measurements. 

### 3.4. Discussion

It has been shown that the technique presented in this work increase the curvature sensitivity by 3.5 times as compared to the original sensor. At this point, as shown in Table 1, it is convenient to compare this enhanced sensitivity with the sensitivities reported by other authors.

The sensitivities reported by sensors 3, 6, and 7 are lower than the sensitivity obtained by our sensor. The sensitivities reported by sensors 1, 2, 4, and 5 are higher than the sensitivity of our sensor, but their curvature range is lower than ours. We should highlight that the sensor 5 (based on ARROW effect) has a high sensitivity, but only measures large curvatures within a narrow curvature range. Based on these parameters, this sensor will not exhibit good performance for structural health monitoring applications where low curvatures are measured [30]. The sensor 3 shows a similar curvature range, but it requires further processing for the taper and has a lower sensitivity. Therefore, our ARROW sensor with PDMS is a good candidate for curvature sensing and has a potential application in structural health monitoring.

We would like to highlight that the CHCF was not modified, and we did not use any special fabrication process rather than coating a section of the CHCF with PDMS. The above simplifies the fabrication process as compared to some of the reported sensors from Table 1. Additionally, although the curvature sensor is embedded in a high TOC polymer, the curvature measurements are not significantly affected by temperature fluctuations. Based on the above results, we can foresee some areas of opportunity for improvement. For instance, since the RI of PDMS at 1550 nm is 1.42, we could further enhance the sensitivity by using a polymer with a higher RI. In this case, an upper limit for the RI will be dictated since we must have ARROW operation. We also believe that applying our technique to the ARROW sensors previously reported in Table 1 can significantly improve their sensitivity as well as their measurable range, according to our results.

## 4. Conclusions

In summary, a cost-effective and reproducible technique to enhance the curvature sensitivity of a curvature sensor based on ARROW guidance was presented and experimentally demonstrated. The fabrication process of the curvature sensor is relatively simple since we just have to splice a segment of CHCF between two SMF. The sensitivity of the curvature sensor is then enhanced by just covering a specific length of the CHCF with PDMS. This simple process eliminates the need for any special procedure or modification of the CHCF to enhance its sensitivity. The curvature sensitivity of the sensor was increased to −5.62 dB/m^−1^, within a sensing range from 0 to 2.68 m^−1^, by covering half of the CHCF with PDMS. Additionally, the PDMS covered sensor exhibited a low-temperature sensitivity of 0.038 dB/°C, showing that temperature changes due to the environment do not alter the curvature values obtained with the device. 

## Figures and Tables

**Table 1:** Comparison of the parameters of our sensor with the reviewed literature.

#	Structure	Sensitivity (dB/m^−1^)	Bending Range (m^−1^)	Article
1	SMF-MMF-SMF	−130.37	From 0.11 to 0.34 (0.23)	[45]
2	Tapered PCF	8.35	From 0.87 to 1.34 (0.47)	[42]
3	SMF-HCF-SMF using an abrupt taper	5.05	From 0.765 to 3.423 (2.65)	[43]
4	Bragg grating in a seven-core fiber	−7.27	From 0 to 1 (1)	[10]
5	SMF-HCF-SMF using ARROW	−15.33	From 3.63 to 4.69 (1.06)	[28]
6	SMF-HCF-SMF using up-taper (ARROW)	−4.28	From 10.72 to 11.6 (0.88)	[33]
7	SMF-HCF-SMF usingARROW	−3.414	From 0 to 2.122 (2.122)	[34]
**8**	**Our work** (SMF-CHCF-SMF using ARROW)	**−5.62**	From 0 to 2.68 **(2.68)**	
